# Endovascular Embolization in Neurovascular Disease: Material Science, Multimodal Management, and Future Horizons

**DOI:** 10.3390/biomedicines14071610

**Published:** 2026-07-17

**Authors:** Thomas Corrado, Wesam Andraous, Sofia Geralemou, Stephen A. Probst, Weidong Wang, Ana Costa

**Affiliations:** Department of Anesthesiology, Renaissance School of Medicine at Stony Brook University, Stony Brook, NY 11794, USA; thomas.corrado@stonybrookmedicine.edu (T.C.); wesam.andraous@stonybrookmedicine.edu (W.A.); sofia.geralemou@stonybrookmedicine.edu (S.G.); stephen.probst@stonybrookmedicine.edu (S.A.P.); weidong.wang@stonybrookmedicine.edu (W.W.)

**Keywords:** angiography, digital subtraction, central nervous system vascular malformations, embolization, therapeutic, hematoma, subdural, chronic, intracranial arteriovenous malformations, meningeal arteries, meningioma, polymers, robotics

## Abstract

**Background & Objectives:** Endovascular embolization has matured into a sophisticated, precision-guided discipline that is central to the management of complex neurovascular pathologies. This review synthesizes contemporary treatment strategies, evaluating the advanced material characteristics of conventional inert liquid polymers, specifically non-adhesive ethylene vinyl alcohol (EVOH) copolymers and adhesive cyanoacrylates, alongside their targeted clinical applications in brain arteriovenous malformations (bAVMs), dural arteriovenous fistulas (dAVFs), hypervascular intracranial tumors, and chronic subdural hematomas (CSDHs). Furthermore, it examines the critical material and hemodynamic constraints that limit these agents in cerebral aneurysm repair. **Methods:** A comprehensive literature synthesis through 3 July 2026 was integrated with peer-reviewed clinical illustrations to evaluate both procedural mechanics and the necessity of post-procedural physiological management. **Review Findings:** Embolization serves a critical dual role: as a definitive curative therapy and as an essential preoperative or radiosurgical adjunct. As demonstrated by recent clinical validations, technical angiographic success must be closely coupled with vigilant neurocritical oversight to manage profound, localized hemodynamic shifts. While these conventional methods represent established clinical practice, the field is evolving away from inert mechanical occlusion toward a highly integrated approach. The convergence of stimuli-responsive “smart” hydrogels and endovascular robotics is being evaluated for potential roles in transforming these interventions into dynamic, bioactive platforms capable of modulating disease-specific mechanisms, such as Rat Sarcoma-Mitogen-Activated Protein Kinase (RAS-MAPK) and Bone Morphogenetic Protein (BMP) signaling in bAVMs or the Von Hippel-Lindau/Vascular Endothelial Growth Factor (VHL/VEGF) axis in hypervascular tumors. This review further analyzes landmark data, including the Squid Trial For the Embolization of the Middle Meningeal Artery for Treatment of Chronic Subdural Hematoma (STEM) trial for CSDH, providing a synthesis for translating these advanced material sciences into standardized, multidisciplinary neurointerventional care.

## 1. Introduction

Embolization is a minimally invasive endovascular procedure designed to block or restrict blood flow to specific anatomical regions, thereby mitigating the risks and physiological symptoms associated with neurovascular disease [1]. The historical trajectory of the field began in 1904, when Robert Dawbarn utilized paraffin and Vaseline to embolize head and neck lesions via the external carotid arteries [2]. However, it was the material science and interventional radiology advancements of the 1970s that triggered the exponential growth in the diversity and efficacy of embolic products available today [3].

Contemporary neurointerventional procedures are undergoing a fundamental shift: we are moving beyond the era of simple mechanical vessel blockage to embrace a new standard defined by bioactive integration and technological synergy. This evolution aims to transform embolic agents from passive fillers into active participants in the healing process. Vascular occlusion can be achieved through the deployment of mechanical devices, such as coils, plugs, and flow diverters or via the injection of particulate and liquid embolic agents [4]. Selection of the appropriate modality is a complex decision-making process involving the analysis of vessel caliber, regional flow dynamics, the intended durability of the occlusion (temporary versus permanent), and the operator’s technical preference [5].

Mechanical devices offer advantages in high-flow environments due to precise placement and minimal risk of non-target embolization. Nevertheless, they are often limited by long procedure times, higher risks of recanalization, and technical difficulty when navigating complex or tortuous anatomy [6]. Complementary to these devices, injectable embolic agents are engineered to flow deep into the vasculature before solidifying. This capability makes them the primary choice for targeting smaller, more distal vessels that are inaccessible to larger mechanical devices [7]. Furthermore, these injectable agents can serve as specialized vehicles for the targeted delivery of drugs [8].

While embolization can serve as a definitive, standalone cure for select vascular lesions, its most frequent and critical role in contemporary neurointervention is as an essential adjunct within a multidisciplinary, multimodal treatment paradigm. Across diverse pathologies, ranging from complex congenital arteriovenous shunts to hypervascular neoplasms, the foundational goal of adjunctive embolization remains consistent: to strategically devascularize the target lesion [9]. In pathologies where surgical resection is necessary, embolization of high-flow arterial feeders and deep-seated microvascular networks prior to resection fundamentally alters local hemodynamics and facilitates surgical success [9,10]. This proactive modification minimizes intraoperative blood loss and improves surgical visualization, thereby enhancing the safety and efficacy of the definitive intervention [10,11].

Despite the rapid expansion of endovascular embolization across diverse neurovascular pathologies, the literature remains highly fragmented. This fragmentation obscures the critical distinctions between established clinical paradigms, the inherent limitations of contemporary inert materials, and the trajectory of emerging technologies. Consequently, the research problem this review addresses is the pressing need to systematize the current evidence base and clearly delineate the developmental trajectory of the discipline. By synthesizing contemporary data across arteriovenous malformations (bAVMs), dural arteriovenous fistulas (dAVFs), hypervascular tumors, and chronic subdural hematomas (CSDHs), this review aims to critically evaluate current multimodal devascularization strategies. Furthermore, it identifies the mechanical and biological constraints of existing liquid embolic agents, establishing the rationale for the field’s necessary evolution toward stimuli-responsive biomaterials and robotic-assisted precision neurointervention.

## 2. Methods

### 2.1. Literature Search Strategy

A comprehensive literature search was conducted to identify relevant studies published from inception through 3 July 2026. The primary electronic databases queried included PubMed/MEDLINE, Scopus, and Embase. To ensure a robust capture of the literature, the search strategy utilized a combination of NIH Medical Subject Headings (MeSH) and free-text keywords. Principal search terms included, but were not limited to: “Embolization, Therapeutic,” “Intracranial Arteriovenous Malformations,” “Central Nervous System Vascular Malformations,” “Hematoma, Subdural, Chronic,” “Ethylene Vinyl Alcohol,” “Cyanoacrylates,” “Hydrogels,” and “Robotics.”

### 2.2. Study Selection Criteria

Two independent reviewers screened the identified articles based on title and abstract, followed by a full-text review to determine eligibility. Inclusion criteria encompassed peer-reviewed clinical trials, prospective and retrospective observational studies, major case series, and highly relevant preclinical or translational studies focusing on advanced endovascular embolic materials. Disagreements regarding study inclusion were resolved through consensus-based discussion between the two reviewers. Study quality was informally assessed based on adherence to reporting standards for observational and clinical studies. Articles were restricted to the English language. Studies focusing exclusively on peripheral embolization, as well as single-patient case reports lacking novel material science or hemodynamic insights, were excluded to maintain the neurovascular focus of this review.

### 2.3. Data Extraction and Synthesis

Given the broad scope of neurovascular pathologies and the heterogeneity of the embolic interventions evaluated, a narrative synthesis approach was employed. Data regarding material science properties, procedural techniques (e.g., transarterial vs. transvenous approaches), multimodal clinical outcomes, and post-procedural physiological management were systematically extracted and evaluated. All citations and duplicate records were managed utilizing EndNote 21.5 software. In instances where multiple datasets existed, preference was given to randomized controlled trials (e.g., STEM, EMBOLISE) and large-scale, multicenter systematic reviews, as these provide the most robust evidence base for establishing current clinical benchmarks and guiding multidisciplinary treatment protocols.

## 3. Embolic Agents: Advanced Material Characteristics

The selection of an embolic agent is governed by the lesion’s angioarchitecture, flow velocity, and the required depth of penetration.

### 3.1. Conventional Liquid Embolic Systems: Ethylene Vinyl Alcohol (EVOH) vs. Cyanoacrylates

Modern liquid agents are distinguished by their ability to achieve a permanent “cast” within the microvasculature, providing deep-seated occlusion. In the current clinical paradigm, these are categorized into two primary chemical classes: non-adhesive EVOH copolymers and adhesive cyanoacrylates.

#### 3.1.1. Non-Adhesive EVOH Copolymers

EVOH is a synthetic, non-adhesive copolymer composed of ethylene and vinyl alcohol units. In neurointerventional applications, EVOH is dissolved in the biocompatible DMSO to create a liquid embolic agent. Current systems such as Onyx™ (Medtronic, Irvine, CA, USA), Squid™ (Balt, Montmorency, France), and PHIL™ (MicroVention, Aliso Viejo, CA, USA) function via a precipitation mechanism. When the solvent DMSO diffuses into the blood, the EVOH copolymer solidifies from the outside-in, forming a spongy mass. This unique phase transition allows for prolonged, “plug-and-push” injections, which are critical for filling complex bAVM nidi or dAVF shunts without immediate catheter entrapment [12,13]. However, DMSO is angiotoxic and can induce vasospasm or inflammatory responses [14].

#### 3.1.2. Adhesive Cyanoacrylates

The primary agent in this class is n-butyl cyanoacrylate (n-BCA; e.g., TRUFILL^®^). This adhesive “glue” undergoes a rapid, exothermic polymerization reaction upon contact with ionic substances (blood). The polymerization rate is highly dependent on the ratio of n-BCA to ethiodized oil, requiring precise timing to avoid unwanted adherence to the delivery catheter. Because it polymerizes nearly instantly upon contact with the target, adhesive cyanoacrylates are preferred for high-flow shunts where rapid, permanent stabilization is required [15,16].

### 3.2. Mechanical and Particulate Dynamics

Bioactive Coils: Modern coils, such as the Matrix™ (Stryker, Kalamazoo, MI, USA) or HydroCoil^®^ (MicroVention, Aliso Viejo, CA, USA), utilize poly(glycolide-co-lactide) (PGLA) and expandable hydrogels (copolymer of acrylamide and acrylic acid), respectively, to increase packing density and facilitate neointimal formation, addressing the high recanalization rates historically seen with bare platinum [17,18].

Traditional Polyvinyl Alcohol (PVA) Particles: Derived from a cross-linked polyvinyl alcohol sponge that is mechanically ground or rasped into shards, traditional PVA represents the foundational particulate embolic agent. Unlike the biologically inert casts formed by liquid agents, the primary mechanism of action for PVA relies on an intense, biologically active foreign body response. Upon deployment, the irregular, angular morphology of the particles promotes mechanical wedging within the vessel lumen. This physical occlusion is rapidly followed by aggressive localized vessel wall inflammation, angionecrosis, and the infiltration of multinucleated giant cells, ultimately culminating in permanent fibrosis and thrombosis [19,20]. Due to their irregular size and shape, these particles frequently aggregate, leading to more proximal occlusion than their specified size range might otherwise dictate [8].

Calibrated Microspheres: Unlike irregular polyvinyl alcohol (PVA) particles that may aggregate proximally, tris-acryl gelatin microspheres (e.g., Embosphere^®^ [Merit Medical, South Jordan, UT, USA]) are highly calibrated for precise distal penetration, making them the standard for terminal arteriolar occlusion in hypervascular tumors [21,22].

## 4. Clinical Applications in Neurovascular Pathologies

### 4.1. Brain Arteriovenous Malformations (bAVMs)

Fundamentally, bAVMs are congenital vascular anomalies defined by a nidus: a tangled vascular network where feeding arteries shunt directly into draining veins without an intervening capillary bed [23]. This structural defect creates a high-flow, low-resistance environment. The molecular drivers of this abnormal angiogenesis vary significantly depending on the clinical phenotype. While syndromic bAVMs, such as those associated with Hereditary Hemorrhagic Telangiectasia (HHT), are classically driven by germline loss-of-function mutations in the Bone Morphogenetic Protein (BMP) signaling pathway, particularly in genes like *Endoglin* (*ENG)* or activin A receptor-like type 1 (*ACVRL1)* that disrupt normal vascular endothelial growth [24], contemporary evidence suggests that somatic activating mutations within the RAS-MAPK pathway are prevalent in sporadic bAVMs, although this is an evolving area of investigation [25,26]. Consequently, high arterial pressure is transmitted directly to thin-walled draining veins, often resulting in their rupture and subsequent intracranial hemorrhage. Furthermore, the aggressive shunting of blood through the nidus can reduce perfusion pressure in adjacent healthy brain tissue (a phenomenon known as vascular steal); this, alongside chronic venous hypertension and hemosiderin deposition, contributes to focal neurological deficits, seizures, and headaches [27,28].

Understanding these pathological features is critical for strategic management, as embolization is frequently employed to mitigate high-risk angioarchitectural features and reduce nidal volume prior to definitive intervention [9]. While the Spetzler-Martin (SM) Grade serves as a foundational tool for estimating microsurgical risk based on nidus size, eloquence of adjacent brain tissue, and deep venous drainage, the overarching management of bAVMs requires a highly individualized approach. Treatment selection is not dictated by grading alone; rather, it depends comprehensively on rupture status, patient age and symptoms, specific angioarchitecture (including associated aneurysms and venous drainage patterns), anatomical accessibility, and the comparative morbidity profiles of available multimodal treatments [29].

#### 4.1.1. Hemodynamic Goals of Embolization

While standalone curative embolization is technically feasible for select small, low-grade (SM Grades I–II) lesions with favorable angioarchitecture, the established clinical benchmarks for small bAVMs (<3 cm) remain stereotactic radiosurgery (SRS) alone or microsurgery due to their high cure rates (>90%) with minimal surgical morbidity [30]. For medium-sized lesions (3–4 cm, typically representing Spetzler-Martin Grade III), the therapeutic strategy often requires a tailored, combined approach. Microsurgical resection remains the primary modality for achieving immediate, definitive cure in surgically accessible nidi, while SRS is frequently employed to complete the obliteration of complex remnants in eloquent regions. Ultimately, within a multidisciplinary framework, embolization, utilizing liquid agents such as n-BCA or Onyx, is predominantly and strategically deployed as a vital adjunct to these definitive therapies [31]. Specifically, it serves three targeted hemodynamic objectives:

Pre-surgical Devascularization: In accordance with the multimodal paradigm, embolization is utilized to occlude deep-seated, surgically inaccessible arterial pedicles, thereby diminishing the hemodynamic pressure within the vascular tangle prior to microsurgical resection [30].

Pre-radiosurgical Volume Reduction: For nidal volumes exceeding 4 cm, the probability of achieving a cure with single-fraction SRS alone diminishes significantly. Strategic embolization is employed here to compartmentalize the high-flow components, thereby optimizing the radiation’s therapeutic index while shielding the adjacent healthy brain tissue from necrosis. By obliterating high-flow compartments, the efficacy of the focused radiation dose is enhanced while minimizing the risk of radiation-induced necrosis in surrounding parenchyma [10,32]. However, it must be acknowledged that the role of embolization prior to SRS remains debated, with conflicting data regarding the impact of nidal fragmentation on subsequent radiation targeting, treatment planning, and ultimate obliteration rates [10,32,33].

Palliative Hemodynamic Modification: In inoperable, deep-seated lesions (e.g., brainstem or thalamus), targeted embolization of intranidal or flow-related aneurysms is performed to reduce the acute risk of rupture [30,34].

The strategic selection of these multimodal interventions, based on nidal characteristics and clinical presentation, is summarized in Table 1.

#### 4.1.2. Procedural Illustration: bAVM Embolization and Post-Procedural Management

**Figure 1:**
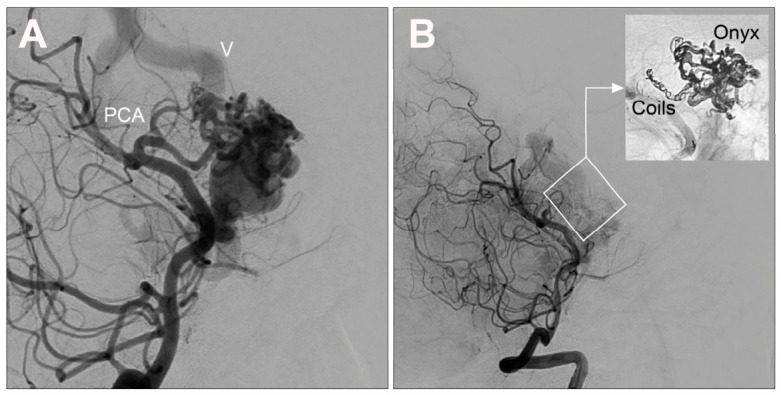
Application of the pressure cooker technique for the endovascular embolization of a complex brain arteriovenous malformation (bAVM). (**A**) Pre-procedural digital subtraction angiography of the vertebral circulation. The imaging outlines the angioarchitecture of the malformation, highlighting the primary arterial supply originating from the posterior cerebral artery (PCA) and its subsequent deep venous drainage (V). (**B**) Post-intervention angiogram confirming near-total obliteration of the vascular nidus. The high-magnification inset details the transarterial embolic configuration, displaying the strategic deployment of microcoils within the feeding pedicle. This coil mass acts as a rigid mechanical barrier against retrograde reflux, ensuring the targeted, high-pressure forward permeation of the Onyx liquid embolic agent into the nidus. [Adapted from Figure 2 in Chen, X.; Wang, Y.; Yu, J. [37] Front. Neurol. 2022, under the Creative Commons Attribution 4.0 International License (CC BY)]. Abbreviations: PCA, posterior cerebral artery; V, venous drainage.

The critical role of liquid embolization as an adjunct to definitive surgical resection or radiosurgery is heavily reliant on the deep, progressive permeation of EVOH-based agents into the complex microvascular architecture of the bAVM. The standard endovascular approach involves diagnostic angiography to clearly identify the high-flow nidus, the dominant arterial feeders, and the rapid venous drainage pathways (Figure 1A). Following super selective microcatheterization into the primary feeding pedicle (Figure 1B), lower-viscosity agents such as Onyx-18 are frequently utilized [37]. To prevent proximal reflux during these prolonged injections, operators may employ the “pressure cooker technique,” deploying microcoils within the feeding pedicle to create a mechanical barrier, as detailed in the magnified inset, prior to Onyx casting. The unique “outside-in” precipitation mechanism of Onyx allows for prolonged, carefully controlled injections. This technique facilitates the targeted opacification of the nidus while minimizing the risk of immediate catheter entrapment.

While this technique is highly effective in achieving a dense embolic cast that reduces nidal turgor and intraoperative blood loss prior to definitive microsurgery, this sudden angiographic obliteration carries inherent, severe risks. As highlighted in recent comprehensive analyses of Onyx embolization, the procedure carries a documented risk of acute intra- and post-operative hemorrhagic complications [37]. Notably, high-pressure casting is a primary driver of these events, accounting for up to 76.5% of intraoperative hemorrhages. Furthermore, operators must navigate this delicate balance by meticulously preserving venous outflow during the initial stages of injection; early or premature occlusion of the primary draining vein drastically increases intranidal resistance and exponentially elevates the risk of intraoperative rupture.

Following significant arteriovenous shunt reduction or complete nidus obliteration, strict post-procedural hemodynamic management is critical to mitigate the risk of Normal Perfusion Pressure Breakthrough (NPPB) and subsequent intraparenchymal hemorrhage [37]. However, rather than adhering to rigid, universally prescriptive blood pressure thresholds, which can inadvertently precipitate cerebral hypoperfusion or ischemia in patients with chronic baseline hypertension, contemporary clinical management emphasizes individualized hemodynamic protocols. Standard endovascular practice typically targets strict normotension or a tailored, modest reduction from the patient’s baseline systolic blood pressure based on clinical stability and individual hemodynamic requirements for the first 24 to 48 h post-procedure. This personalized target must be dynamically balanced against the extent of acute devascularization, the patient’s baseline vascular resistance, and serial neurological examinations to safely navigate the therapeutic window between hyperperfusion hemorrhage and ischemic insult [28,37,38].

### 4.2. Dural Arteriovenous Fistulas (dAVFs)

Dural arteriovenous fistulas (dAVFs) represent an acquired pathology characterized by the shunting of arterial blood directly into dural veins or venous sinuses. Unlike congenital arteriovenous malformations, most dAVFs are thought to be triggered by physiological stressors such as trauma, prior surgery, venous stenosis, or dural sinus thrombosis. These events likely initiate a cycle of local venous hypertension and subsequent neoangiogenesis, leading to the recruitment of meningeal arterial branches that bypass the normal capillary bed [39]. In dAVFs, this process is often driven by Notch signaling upregulation, which governs arterial–venous differentiation in response to chronic venous hypertension [40,41].

The clinical severity of a dAVF is determined by its venous drainage pattern. Utilizing the Cognard and Borden systems, “aggressive” phenotypes (Cognard III/IV, Borden III) are defined by the presence of cortical venous reflux (CVR), where high-pressure arterial flow is diverted into cortical veins. This retrograde flow results in venous hypertension, which carries a significant annual hemorrhagic risk of 8.1% and a mortality rate of 10.4% [42]. Consequently, the therapeutic goal focuses on the permanent obliteration of the fistulous point to normalize venous hemodynamics and eliminate the risk of catastrophic rupture [39].

#### 4.2.1. Procedural Strategy: TAE vs. TVE

Transarterial Embolization (TAE): Leveraging EVOH agents, TAE is often first-line. The goal is to navigate the microcatheter as close to the shunt as possible, allowing the embolic agent to cross the fistulous point and partially fill the proximal venous outlet. This “cast” ensures the shunt is truly obliterated and cannot be recruited by collateral arterial supply [43].

Transvenous Embolization (TVE): TVE is highly effective for cavernous sinus dAVFs or lesions involving non-functional, thrombosed sinuses. However, TVE is strictly contraindicated if the involved sinus maintains exclusive or irreplaceable normal venous drainage. Its occlusion could precipitate catastrophic venous infarction. To definitively mitigate this risk, intraoperative adjuncts such as balloon test occlusion or venous manometry can be utilized to confirm sinus functional status and evaluate collateral venous capacity before committing to the permanent deployment of liquid embolic agents [44].

#### 4.2.2. Multimodal Salvage and Synergy

The management of complex dAVFs frequently requires the coordinated integration of endovascular, surgical, and radiosurgical modalities to ensure complete obliteration of the shunt.

Preoperative Adjunct: For anatomically challenging, high-flow tentorial or petrosal dAVFs, TAE is strategically deployed to reduce arterial inflow and venous congestion prior to definitive microsurgical disconnection [45].

Microsurgical Disconnection: Surgery remains the definitive rescue strategy for dAVFs located in anatomically challenging regions, such as the anterior cranial fossa or ethmoidal junction, where arterial access is often unsafe, prohibitive, or excessively tortuous, offering immediate angiographic cure rates of >95% [46].

Stereotactic Radiosurgery: SRS functions as a secondary “clean-up” mechanism targeted at small, residual shunts characterized by low-risk venous drainage. Due to its inherent 1–3 year latency period, SRS is generally excluded as a primary intervention for high-grade lesions with active cortical venous reflux (CVR), as these patients require immediate protection against hemorrhagic events [47].

The synergistic application of these techniques within a specialized neurovascular team represents the contemporary clinical benchmark, optimizing efficacy and providing durable outcomes for complex lesions. Ultimately, because venous drainage architecture and access feasibility vary drastically by cranial region, the strategic sequencing of these endovascular, surgical, and radiosurgical interventions is fundamentally dictated by the specific anatomical location of the fistula. A comprehensive breakdown of these tailored multimodal strategies across distinct high-risk dAVF locations is detailed in Table 2.

#### 4.2.3. Clinical Illustration: Tentorial dAVF Embolization Using a Coil-Assisted Anti-Reflux Technique

The clinical utility of advanced endovascular techniques is powerfully exemplified in the management of complex, high-risk lesions such as tentorial dural arteriovenous fistulas (dAVFs). These lesions frequently present with aggressive clinical features, such as cerebellar hemorrhage, and often recruit a complex arterial supply from deep, tortuous meningeal networks, including the meningohypophyseal trunk (MHT) and the medial tentorial artery (MTA) of Bernasconi–Cassinari. Achieving comprehensive embolic penetration of the fistulous point while protecting adjacent, normal neurovasculature requires highly precise flow-control strategies.

**Figure 2:**
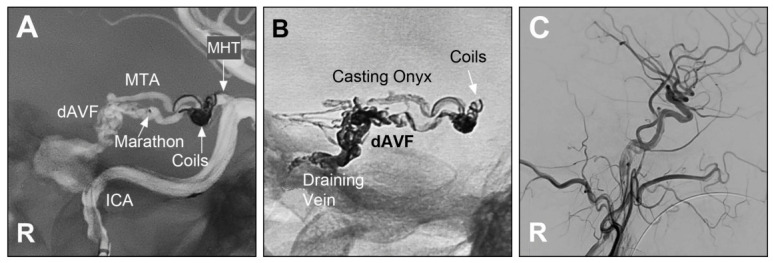
Endovascular embolization of a tentorial dural arteriovenous fistula (dAVF) utilizing a coil-assisted anti-reflux technique. (**A**) Procedural roadmap demonstrating the transarterial setup. A Marathon™ microcatheter (Medtronic, Irvine, CA, USA) is navigated into the medial tentorial artery (MTA), while a protective coil mass is deployed proximally within the meningohypophyseal trunk (MHT) to establish a mechanical plug. (**B**) Unsubtracted fluoroscopic imaging during the injection phase. The proximal coil mass successfully arrests the retrograde reflux of the Onyx liquid embolic, forcing the agent antegrade to comprehensively permeate the dAVF and the proximal draining vein. (**C**) Final post-procedural digital subtraction angiogram of the right internal carotid artery (ICA) confirming complete angiographic obliteration of the fistula. [Adapted from Figure 2 in Hou, K.; Yu, J. [48] Front. Neurol. 2022, under the Creative Commons Attribution 4.0 International License (CC BY)]. Abbreviations: dAVF, dural arteriovenous fistula; ICA, internal carotid artery; MHT, meningohypophyseal trunk; MTA, medial tentorial artery; R, right.

As demonstrated in the recent literature, a transarterial approach utilizing a dual-microcatheter, coil-assisted anti-reflux technique provides exceptional control when deploying Onyx in these challenging anatomies [48]. In a representative case of a patient presenting with a ruptured tentorial dAVF (Figure 2A), an embolization microcatheter was navigated super-selectively into the MTA, while a secondary microcatheter was placed proximally within an aneurysmal dilation of the MHT.

Crucially, prior to liquid embolization, a protective coil mass was deployed within the MHT via the secondary catheter. This established a robust mechanical barrier or “plug.” During the active injection phase (Figure 2B), this proximal coil mass successfully arrested any retrograde reflux of the Onyx liquid embolic. By eliminating the path of least resistance backward along the catheter shaft, the agent was forced antegrade, driving deeper to comprehensively permeate the dAVF nidus and the proximal draining vein. The final post-procedural digital subtraction angiogram (Figure 2C) confirms complete, durable angiographic obliteration of the fistula with no residual high-flow shunting.

This case demonstrates the definitive curative potential of transarterial liquid embolization using advanced mechanical adjuncts, particularly when navigating anatomies with a high risk of embolic reflux. However, the sheer anatomical diversity of dAVFs often dictates the tailored, combined-therapy approaches previously outlined in this review.

### 4.3. Hypervascular Intracranial Tumors

Many aggressive intracranial tumors, including primary meningiomas and systemic metastases, are defined by their ability to recruit a robust, neoplastic arterial supply. The resulting tumor-specific neovascular networks exhibit strikingly abnormal features, most notably high-flow arteriovenous shunting, disorganized endothelial architecture, and increased capillary permeability [49]. Given this fragile and extensive vascularity, preoperative embolization is frequently deployed to selectively prune the tumor’s arterial supply, thereby mitigating the profound surgical risks of obscured dissection planes and hemorrhage [11].

#### 4.3.1. Tumor-Specific Angioarchitecture and Molecular Drivers

The specific angioarchitecture and molecular drivers of this vascular proliferation vary significantly across different intracranial tumor subtypes.

Meningiomas and Complex Skull Base Tumors: Meningiomas are dural-based tumors that typically recruit their vascular supply from the middle meningeal artery (MMA) and other branches of the external carotid artery (ECA). Similarly, juvenile nasopharyngeal angiofibromas (JNAs) and paragangliomas often exhibit extensive recruitment of the internal maxillary artery alongside other complex ECA networks [50].

Renal Cell Carcinoma (RCC) Metastases: In contrast, systemic metastases like renal cell carcinoma (RCC) and melanoma exhibit aggressive arterial recruitment. In RCC, this angiogenic drive is frequently initiated by Von Hippel–Lindau (*VHL*) gene inactivation, which constitutively stabilizes hypoxia-inducible factors and massively upregulates Vascular Endothelial Growth Factor (VEGF) [51]. Consequently, RCC often recruits both meningeal and pial feeding vessels, necessitating aggressive devascularization strategies utilizing permanent agents like EVOH or PVA.

Melanoma Metastases: Parallel to this, metastatic melanoma frequently harbors *BRAF* gene mutations that elevate the risk of aggressive brain involvement. Its vascularity is highly heterogeneous; some lesions are not highly arterial, making the clinical benefit of embolization case-dependent [52].

The specific clinical roles of various embolic agents, alongside the comparative advantages of devascularization strategies for these neoplasms, are summarized in Table 3.

#### 4.3.2. The Anatomy of “Dangerous Anastomoses”

The foremost procedural risk in tumor embolization is the existence of extracranial-to-intracranial (external carotid artery to internal carotid artery/vertebral artery, or ECA-to-ICA/VA) anastomoses.

Non-Target Ischemia: Inadvertent migration of embolic material through these channels can precipitate ischemic stroke or cranial nerve palsies [56].

Common High-Risk Sites: Crucial anastomotic sites include the ophthalmic artery (via the ethmoidal branches), the inferolateral trunk (ILT), and the neuromeningeal branches of the ascending pharyngeal artery supplying the lower cranial nerves [57].

Mitigation Strategy: Successful devascularization requires superselective catheterization and a detailed understanding of skull base vascular variants to ensure the embolic cast remains isolated within the tumor bed [58,59].

#### 4.3.3. Clinical Illustration: Standalone Embolization of a Hypervascular Meningioma

While the clinical utility of endovascular therapy for intracranial neoplasms is traditionally viewed as an adjunct to microsurgery, advancements in liquid embolic agents and microcatheter technologies have enabled the exploration of standalone embolization for highly selected hypervascular tumors. This minimally invasive paradigm is particularly relevant for small, asymptomatic meningiomas supplied exclusively by the ECA system, where aggressive surgical resection may carry disproportionate morbidity or patient preference dictates conservative management.

**Figure 3:**
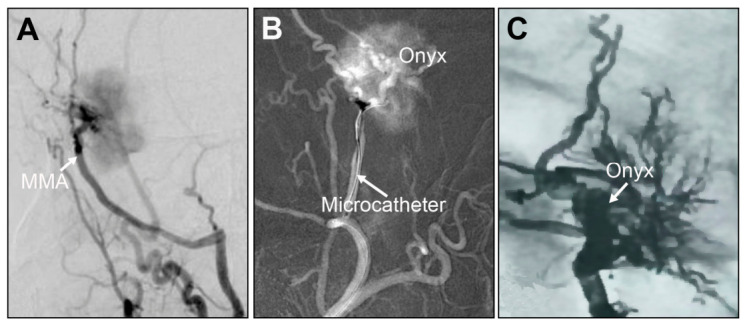
Standalone endovascular embolization of a hypervascular intracranial meningioma via the ECA system. (**A**) Pre-procedural digital subtraction angiogram demonstrating a robust tumor blush. The white arrow denotes the primary arterial supply originating from the MMA. (**B**) Intraoperative fluoroscopy illustrating super-selective navigation, with white arrows highlighting the microcatheter positioned within the feeding pedicle and the initial permeation of the Onyx-18 liquid embolic agent into the tumor vascular bed. (**C**) Final post-embolization angiographic cast. The white arrow indicates the dense Onyx accumulation, confirming extensive (~90%) devascularization of the meningioma without the need for open craniotomy. [Adapted from Figure 1 in Yu, D., et al. [53] Front. Oncol. 2025, under the Creative Commons Attribution 4.0 International License (CC BY)]. Abbreviations: ECA, external carotid artery; MMA, middle meningeal artery.

As demonstrated in recent multicenter evaluations, standalone embolization can achieve durable radiographic stabilization or active volume regression in these select cohorts [53]. In a representative case of a patient presenting with an incidentally discovered right sphenoidal ridge meningioma (Figure 3A), cerebral angiography confirmed a robust, singular arterial supply originating from the MMA.

Utilizing a superselective technique, a microcatheter was navigated into the distal feeding pedicle (Figure 3B), followed by the controlled, progressive injection of the liquid embolic agent Onyx-18. The penetration of the liquid embolic allowed for a dense, total angiographic devascularization, obliterating approximately 90% of the tumor’s microvascular bed without the need for open craniotomy (Figure 3C). Long-term imaging in such cases frequently demonstrates subsequent tumor necrosis and volume reduction, underscoring the emerging potential of standalone liquid embolization as a definitive, minimally invasive alternative for highly selected meningiomas with favorable angioarchitecture.

### 4.4. Chronic Subdural Hematomas (CSDHs)

The pathogenesis of a chronic subdural hematoma (CSDH) is defined by the gradual accumulation of blood and fluid within the space between the dura mater and the arachnoid membrane, typically occurring in the weeks following a mild head injury that causes the slow rupture of bridging veins. This pathology predominantly affects the elderly, individuals on anticoagulant therapy, and those with pre-existing brain atrophy [60,61]. The expansion of the hematoma is driven by a complex cycle of chronic inflammation and aberrant angiogenesis. As the initial blood collection lyses, it creates an osmotic gradient that draws in additional fluid. Concurrently, the surrounding hematoma membrane develops a network of fragile, hyperpermeable macrocapillaries supplied by the MMA, which continuously leak plasma and blood into the subdural space [61,62]. This progressive expansion exerts a mass effect on the brain, leading to diverse neurological symptoms such as cognitive decline, persistent headaches, focal motor deficits, or seizures.

#### 4.4.1. Rationale and Outcomes of MMA Embolization

Conventional surgical evacuation addresses immediate fluid collection but ignores the underlying vascular leakage; thus, CSDH management has increasingly shifted toward MMA devascularization [63]. Endovascular occlusion of these hyperpermeable neo-capillaries interrupts the chronic inflammatory exudation cycle, directly treating the hematoma’s pathophysiology [64]. While liquid embolic agents provide robust dural permeation, PVA particles have emerged as a highly favored and cost-effective alternative for MMA devascularization. Functioning as flow-directed agents, PVA particles are passively carried by the dominant hemodynamics directly into the capillary networks of the hematoma membrane. Furthermore, whereas liquid agents necessitate precise microcatheter positioning to manage rapid polymerization and solvent-related toxicity, PVA safely achieves deep, terminal arteriolar occlusion through distal mechanical wedging [65]. Strategic particle sizing (typically 150–350 μm) is paramount: particles must be small enough to penetrate the aberrant dural micro-network yet large enough to prevent inadvertent shunting through high-risk extracranial-to-intracranial anastomoses [64]. Additionally, the use of PVA circumvents the need for specialized, DMSO-compatible microcatheters, reducing the logistical complexity of the intervention [66]. Clinically, MMA embolization addresses the underlying pathophysiology, reducing historical recurrence rates from 20% to 2–10% [67].

While particulate agents such as PVA have historically served as the cornerstone for reducing flow through meningeal networks, their lack of cohesive solidification limits their utility in achieving deep, permanent occlusion of fragile, high-pressure vascular beds. This limitation has driven a significant shift toward the utilization of liquid embolic agents. Unlike particulates, which act by physical obstruction, modern injectable agents are engineered to transition from a liquid to a solid state in situ, allowing for controlled, progressive penetration into the distal capillary networks. This transition, from simple mechanical flow restriction to comprehensive angioarchitectural obliteration, is best illustrated in the treatment of chronic subdural hematomas (CSDHs), where liquid embolic agents are now being leveraged to neutralize the pathological neovascular membranes that drive hematoma recurrence.

#### 4.4.2. Clinical Illustration: MMA Embolization for Chronic Subdural Hematoma

The management of chronic subdural hematomas (CSDHs) has recently undergone a massive paradigm shift, driven by the understanding that pathological neovascular membranes are responsible for recurrent micro-hemorrhages and ongoing hematoma expansion. MMA embolization has rapidly emerged as a transformative intervention designed to directly devascularize these fragile capillary networks, thereby promoting hematoma resorption and preventing recurrence.

**Figure 4:**
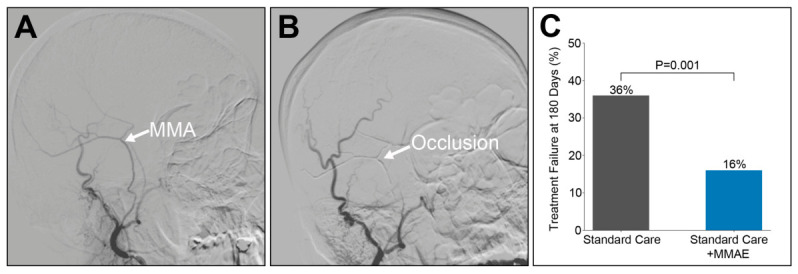
Procedural and clinical validation of MMA embolization for Chronic Subdural Hematoma (CSDH). (**A**) Selective right MMA angiography demonstrating pathological neovascular supply to the CSDH prior to intervention. The white arrow denotes the primary arterial supply originating from the MMA. (**B**) Post-procedural angiography confirming complete obliteration of the abnormal meningeal branches following the injection of a liquid embolic agent, with the white arrow indicating the point of vessel occlusion. (**C**) Impact of adjunctive MMA embolization on overall treatment failure. Data derived from the STEM trial demonstrates a significant reduction in treatment failure, evaluated as a composite metric of required surgical rescue, lack of hematoma resolution, or major disabling neurological events, when endovascular devascularization is combined with standard care (16% vs. 36%; *p* = 0.001). [Panels A and B adapted from Soetanto & Wiyarta [68], Neurol. Int. 2026, under the Creative Commons Attribution 4.0 International License (CC BY). Panel C data sourced and graphed from Fiorella et al. [69], N Engl J Med, 2025]. Abbreviations: CSDH, chronic subdural hematoma; MMA, middle meningeal artery; MMAE, middle meningeal artery embolization; STEM, Squid Trial for the Embolization of the Middle Meningeal Artery for Treatment of Chronic Subdural Hematoma.

The procedural execution of this technique relies on excellent distal penetration, as demonstrated in the recent literature [68]. In a representative case of a patient presenting with a massive right-sided CSDH resulting in marked cortical compression and subfalcine herniation, standalone MMA embolization was pursued. Selective angiography of the right MMA (Figure 4A) clearly delineates the robust, pathological neovascular supply feeding the hematoma capsule. Utilizing a transradial approach, operators superselectively catheterized the distal MMA branches and deployed the liquid embolic agent n-BCA. Post-procedural angiography (Figure 4B) confirms the complete and durable obliteration of the abnormal meningeal supply, effectively neutralizing the source of recurrent bleeding.

Beyond technical feasibility, the clinical efficacy of this endovascular approach has now been rigorously validated by high-level, randomized controlled data. As demonstrated by the pivotal Squid Trial for the Embolization of the Middle Meningeal Artery for Treatment of Chronic Subdural Hematoma (STEM), the addition of MMA embolization to standard care dramatically alters the disease course [69]. Quantitative analysis (Figure 4C) confirms a highly significant reduction in overall treatment failure, evaluated as a composite metric of required surgical rescue or a lack of hematoma resolution, when compared to standard care alone (16% vs. 36%; *p* = 0.001). This robust validation solidifies targeted endovascular devascularization as a critical, front-line component in modern neurotrauma management.

Recent major randomized controlled trials, most notably the STEM trial, have validated MMA embolization as a highly effective strategy to reduce CSDH recurrence and progression [69,70,71]. By devascularizing the fragile, highly permeable neovessels within the subdural membranes, endovascular embolization effectively arrests the continuous micro-hemorrhages driving hematoma growth. However, a critical clinical distinction must be maintained regarding patient selection and mass effect. MMA embolization does not provide immediate mechanical decompression; rather, subsequent hematoma resorption is a gradual, physiological process occurring over weeks to months. Consequently, while standalone MMA embolization represents a potential primary therapeutic option for carefully selected, clinically stable patients with minimal mass effect, it is strictly insufficient as a standalone intervention for patients presenting with severe midline shift, brainstem compression, or acute neurological deterioration. In these high-risk clinical scenarios, immediate surgical evacuation (e.g., burr hole craniostomy or craniotomy) remains the mandatory primary standard of care to rapidly relieve intracranial pressure, with MMA embolization utilized selectively as an adjunctive, prophylactic measure to mitigate the risk of post-operative recurrence [72].

### 4.5. Cerebral Aneurysms: Contemporary Management and the Constraints of Liquid Agents

The pathogenesis of cerebral aneurysms is a complex process driven by the interplay of aberrant hemodynamics, localized inflammation, and extracellular matrix remodeling. Elevated wall shear stress at arterial bifurcations disrupts endothelial barrier function, recruiting circulating inflammatory cells [73]. The resulting release of pro-inflammatory cytokines and hyperactivation of matrix metalloproteinases systematically degrade structural proteins, fragmenting the internal elastic lamina and load-bearing collagen networks [74]. This progressive structural degradation results in a fragile, thin-walled aneurysm sac that is exceptionally vulnerable to rupture [75,76,77].

#### 4.5.1. The Contemporary Standard of Care and Historical Context

Due to this extreme morphological fragility, the contemporary endovascular standard of care is heavily dominated by mechanical devices. Endovascular coiling (both bare platinum and bioactive variants) remains a foundational therapy, while flow-diverting stents and newer intrasaccular flow disrupters (e.g., the Woven EndoBridge [WEB] device) have revolutionized the treatment of complex and wide-necked aneurysms [78,79,80]. These mechanical modalities achieve intra-aneurysmal thrombosis and eventual endothelialization without introducing severe thermal or chemical stressors.

Historically, there was clinical experience utilizing highly viscous liquid agents, such as Onyx HD-500, specifically for the treatment of complex, wide-necked aneurysms [81,82,83]. To mitigate the risks of non-target embolization, these procedures relied heavily on device-assisted delivery, notably the balloon-remodeling technique, to temporarily protect the parent artery during the prolonged injection and precipitation phases. However, this approach saw limited widespread adoption and was largely superseded by flow diversion due to the high technical complexity of the procedure, prolonged procedural times, the requirement for temporary flow arrest, and the inherent angiotoxic profile of the DMSO solvent.

#### 4.5.2. The Constraints of Contemporary Liquid Agents

Consequently, while modern liquid agents are essential for nidal lesions, they are rarely indicated as primary therapy for cerebral aneurysms today due to two major safety constraints:

Hemodynamic and Thermal Stress: The polymerization of n-BCA is highly exothermic, and the DMSO solvent utilized in EVOH systems is angiotoxic. Both properties can trigger acute rupture in these already thin-walled or dissecting aneurysms, inducing localized wall tension and potential thermal injury during the phase transition of the embolic agent [84,85].

Morphological Preservation: The primary goal in aneurysm treatment is complete sac occlusion with the strict preservation of the parent artery. The unpredictable “free-flow” nature of liquid embolic agents carries a prohibitive risk of reflux into the parent vessel, potentially necessitating emergent bypass surgery [86,87].

#### 4.5.3. Evolving Toward Emerging Injectable Biomaterials

The inability to safely deploy current liquid agents within fragile, degraded aneurysmal sacs exposes the fundamental limitation of contemporary embolotherapy: it relies on inert, physically occlusive, and often biologically harsh materials.

To overcome these dual challenges of hemodynamic instability and procedural angiotoxicity, the field must evolve beyond simple mechanical blockage. The recent literature has increasingly focused on emerging injectable biomaterials designed specifically for the delicate aneurysmal microenvironment. Beyond stimuli-responsive “smart” hydrogels, contemporary studies are investigating shear-thinning biomaterials, non-DMSO-based precipitating polymers, and bioresorbable shape-memory foams. These advanced materials aim to provide immediate, cohesive sac occlusion without exothermic stress, actively mitigating vascular stress and promoting localized cellular healing to set the foundation for the next generation of precision neurointerventions [88,89]. The specific mechanistic profiles of these smart biomaterials, along with the endovascular robotic platforms required for their precision delivery, are explored further in Section 5.

## 5. Future Perspectives: Toward Precision Neurointervention

The field of neurovascular embolization is rapidly transitioning from a discipline defined by mechanical occlusion to one driven by molecular biology and advanced endovascular robotics. Future innovations aim to move beyond creating inert physical casts, leveraging bioactive scaffolds as dynamic platforms to actively guide cellular healing and utilizing robotics to achieve unprecedented procedural precision.

### 5.1. Molecularly Targeted Embolotherapy

The next generation of embolic materials is envisioned to function simultaneously as occlusive agents and localized delivery vehicles for targeted molecular therapeutics.

Anti-Angiogenic Elution and Pathway Modulation: In vitro studies suggest that for aggressive dAVFs and hypervascular tumors, future embolic matrices could be conceptualized to elute localized endothelial inhibitors, such as VEGF antagonists, directly into the nidus to halt post-procedural neoangiogenesis at the receptor level [86,87]. A primary biophysical challenge in these high-flow arteriovenous shunts is preventing the rapid hemodynamic washout of these therapeutics [90]. Overcoming this requires advanced polymer configurations, such as stimuli-responsive interpenetrating network hydrogels, that uncouple the physical macro-occlusion from the drug release kinetics [91]. By tethering molecular antagonists via enzymatically cleavable linkers or utilizing controlled-degradation microspheres, these scaffolds are projected to maintain sustained, localized inhibition despite severe local shear stress [91,92]. Furthermore, drawing upon non-human in vivo models of hereditary hemorrhagic telangiectasia, therapies could be designed to modulate specific morphogenetic signaling cascades, such as the BMP pathway, which is critically implicated in hereditary hemorrhagic telangiectasia and aberrant vascular development [24,93,94]. Specifically, recognizing that bAVMs often arise from loss-of-function mutations in the ACVRL1/ENG receptor complex, smart biomaterials could ultimately be engineered for the localized elution of recombinant BMP10 or targeted downstream inhibitors, such as Angiopoietin-2 antagonists [95,96]. While primarily validated in non-neurovascular fields and animal models, these localized therapeutics seek to compensate for the deficient SMAD1/5/8 intracellular signaling cascade to actively suppress the hyperproliferative, disorganized vascular phenotype characteristic of bAVM nidi, restoring endothelial quiescence and guiding true physiological remodeling [95,97].

Cellular Reprogramming and Endothelialization: Although currently restricted to the biomaterial research phase, future bioactive scaffolds aim to address underlying cellular pathophysiology by inhibiting the pro-inflammatory, synthetic phenotypic switch of surrounding vascular smooth muscle cells (VSMCs) to instead promote organized, physiological endothelialization [98,99]. Achieving this targeted healing requires scaffolds functionalized with specific bioactive peptide sequences, such as RGD (Arg-Gly-Asp) or YIGSR (Tyr-Ile-Gly-Ser-Arg), designed to selectively engage endothelial cell surface receptors like the α_v_β_3_ and α_5_β_1_ integrins [100,101]. This precise receptor–ligand interaction not only facilitates the recruitment and adhesion of circulating endothelial progenitor cells but also actively suppresses the hyperactivation of matrix metalloproteinases [102,103]. By stabilizing the extracellular matrix at the molecular level, these “smart” agents could hypothetically guide true vascular remodeling rather than functioning as inert mechanical plugs [101].

### 5.2. Endovascular Robotics and Computational Integration

**Figure 5:**
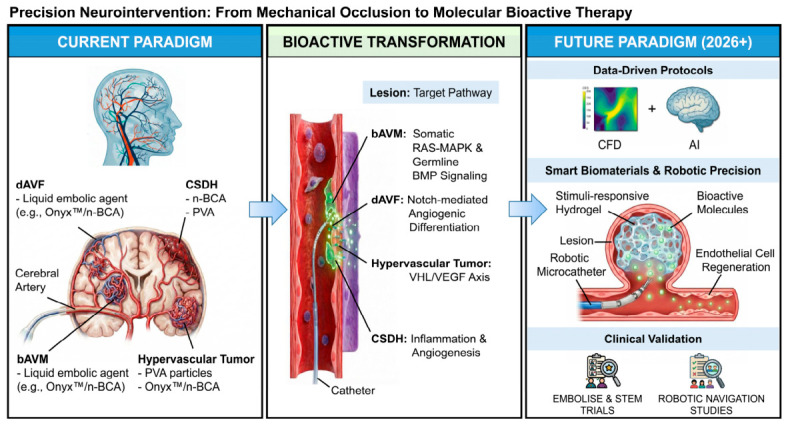
The evolution of endovascular embolization: From macroscopic mechanical occlusion to precision molecular therapy. (**Left**) Current Paradigm: Standard-of-care endovascular management relies on the mechanical occlusion of aberrant vascular networks. Intracranial vascular lesions, including arteriovenous malformations (bAVMs), dural arteriovenous fistulas (dAVFs), hypervascular tumors, and chronic subdural hematomas (CSDH), are currently treated using inert, space-filling materials. Conventional embolic agents, such as liquid polymers (e.g., Onyx™, n-BCA) and polyvinyl alcohol (PVA) particles, act as physical barricades to halt flow and mitigate hemorrhagic risk but lack the capacity for biological integration. (**Center**) Bioactive Transformation: The therapeutic focus is shifting from gross angioarchitecture to the underlying molecular pathophysiology. Next-generation interventions target the distinct developmental and pathological signaling cascades driving lesion genesis. Key molecular targets include the somatic RAS-MAPK pathway and germline BMP signaling in bAVMs, Notch-mediated angiogenic differentiation in dAVFs, the VHL/VEGF axis in hypervascular tumors, and localized inflammation and angiogenesis pathways in CSDHs. (**Right**) Future Paradigm (2026+): The integration of multidisciplinary technologies aims to achieve true vascular remodeling rather than inert blockage. Data-driven protocols utilizing artificial intelligence (AI) and computational fluid dynamics (CFD) guide semi-autonomous, sub-millimeter robotic microcatheter navigation. In situ, stimuli-responsive “smart” hydrogels elute targeted bioactive molecules to actively modulate the local microenvironment and promote endothelial cell regeneration. This transition from mechanical to biological healing is being validated through standardized clinical protocols, including recently completed landmark randomized controlled trials for CSDH (e.g., STEM, EMBOLISE) and investigational robotic navigation studies. Abbreviations: AI, artificial intelligence; bAVM, brain arteriovenous malformation; BMP, bone morphogenetic protein; CFD, computational fluid dynamics; CSDH, chronic subdural hematoma; dAVF, dural arteriovenous fistula; n-BCA, n-butyl cyanoacrylate; PVA, polyvinyl alcohol; RAS-MAPK, Rat Sarcoma-Mitogen-Activated Protein Kinase; VEGF, vascular endothelial growth factor; VHL, Von Hippel-Lindau.

However, the prospective clinical translation of these highly sensitive, targeted molecular therapeutics is contingent upon their precise spatial delivery within complex vascular architectures. While smart biomaterials conceptually offer unprecedented biological precision, the field must simultaneously overcome the mechanical limitations of manual catheter navigation to fully exploit their potential. Consequently, the integration of robotic platforms with predictive computational models aims to fundamentally alter intraoperative capabilities, potentially bridging the critical gap between molecular design and exact anatomical deployment (Figure 5).

Ultra-Distal Precision Navigation: Robotic-assisted systems provide sub-millimeter, tremor-free microcatheter manipulation, essential for navigating highly tortuous neo-networks and deploying liquid agents with steady-state injection pressures [104,105]. This mechanical stability will be uniquely critical when administering the next generation of stimuli-responsive hydrogels, ensuring exact local residence times before in situ crosslinking occurs.

Closed-Loop Hemodynamic Control: When integrated with Computational Fluid Dynamics (CFD), operators can calculate wall shear stress and local perfusion pressures before the embolic cast solidifies, executing staged occlusion strategies that actively prevent normal perfusion pressure breakthrough [106,107,108].

### 5.3. Smart Biomaterials and Active Clinical Trials

To fully leverage robotic precision, embolic agents are evolving into “smart” platforms evaluated through an expanding portfolio of clinical trials designed to validate next-generation platforms.

Stimuli-Responsive Systems: Research into thermoresponsive and ion-sensitive hydrogels includes the Embrace Hydrogel study [109]. While this specific trial is evaluating a polyethylene glycol (PEG)-based system designed for instantaneous in situ crosslinking to treat arterial bleeding in solid organs and peripheral arteries, it serves as vital translational evidence. Successfully adapting these stimuli-responsive “aqueous ionic liquids” to neurovascular application, such as the devascularization of hypervascular tumors, could offer predictable solidification and durable occlusion while avoiding the angiotoxicity associated with DMSO [110,111].

Material Advancements and Bioresorbable Scaffolds: Recent observational studies, such as the multicenter LIQUID study, are assessing low-viscosity EVOH copolymers (e.g., Squid-12) to reduce imaging artifacts and improve distal penetration [13,112]. Concurrently, extracellular matrix (ECM)-derived hydrogels aim to create temporary occlusions that facilitate healing before fully resorbing [113,114].

Protocol Standardization and Automation: Recent landmark Phase III randomized controlled trials, most notably the STEM trial (NCT04410146) and the EMBOLISE trial (NCT04402632), are rigorously validating how targeted devascularization impacts long-term recurrence compared to standard surgical evacuation [71,115].

The Future of Aneurysm Repair: While current validations of endovascular robotic platforms (such as the CorPath GRX system) are primarily focused on achieving tremor-free delivery within fragile neo-networks and preclinical AVM models, their demonstration of semi-autonomous navigation provides a critical technical foundation [116,117]. This existing robotic framework, characterized by sub-millimeter precision, could theoretically transition aneurysm treatment from purely mechanical coiling toward a new paradigm of bioactive sac reconstruction. When paired with next-generation stimuli-responsive hydrogels that lack the exothermic and angiotoxic profiles of current agents, these endovascular robotic platforms offer a viable pathway to overcome the current safety ceiling of aneurysmal repair [85].

### 5.4. Technological Limitations and Barriers to Clinical Adoption

While the integration of bioactive scaffolds, molecularly targeted therapies, and endovascular robotics represents a promising paradigm shift, several inherent limitations and potential barriers to clinical adoption must be acknowledged. The shift from inert physical fillers to bioactive scaffolds faces a universal challenge, irrespective of the specific pathology. Whether managing the high-pressure, congenital shunts of a bAVM or the fragile, acquired neovascular membranes of a CSDH, the fundamental hurdle remains the achievement of controlled, in situ bioactivity without compromising safety or triggering non-target ischemia. Regarding smart biomaterials, the primary challenge remains achieving predictable, highly controlled in situ phase transitions without premature polymerization within the microcatheter or delayed crosslinking that could result in non-target distal embolization. Furthermore, the long-term biocompatibility and degradation profiles of these novel hydrogels in the human neurovascular environment require rigorous longitudinal validation [84,88]. The clinical translation of localized molecular therapies, such as VEGF or Angiopoietin-2 antagonists, is complicated by the extreme hemodynamic shear stress of arteriovenous shunts, which threatens to wash out therapeutic agents before sustained receptor engagement can occur [90,91].

Similarly, the widespread clinical adoption of endovascular robotic platforms faces significant logistical and economic hurdles. The high initial capital expenditure, the need for specialized multidisciplinary training, and the current lack of tactile haptic feedback for the operator present substantial barriers. Furthermore, the requirements for specialized sterilization and complex software maintenance are hereby increasing the logistical complexity of the intervention [104]. Until these platforms can definitively demonstrate a reduction in procedural morbidity that justifies their cost and logistical complexity, their use will likely remain confined to highly specialized, high-volume academic neurovascular centers.

## 6. Limitations of the Present Work

A dedicated discussion of the limitations of the present work is necessary to properly contextualize its findings. First, because this manuscript is structured as a comprehensive narrative review rather than a systematic review or meta-analysis, the literature selection and synthesis are inherently susceptible to selection bias. We did not employ quantitative statistical pooling of complication or obliteration rates across the evaluated modalities. Second, the ambitious scope of this review, spanning bAVMs, dAVFs, hypervascular tumors, CSDH, and cerebral aneurysms, necessitates a broad overview, which inherently limits the critical depth with which any single pathology or specific embolic agent can be evaluated. Finally, the field of neurointerventional material science is evolving rapidly. Because we have intentionally included discussions of preclinical and early-phase translational concepts to outline future trajectories, some of the hypothesized clinical integrations may ultimately fail to demonstrate safety or efficacy in human trials.

## 7. Conclusions

Endovascular embolization has solidified its role as an indispensable, cross-cutting modality within modern neurovascular practice. Currently established clinical applications demonstrate its vital dual function: as a highly effective definitive therapy, notably in the management of aggressive dural arteriovenous fistulas (dAVFs) and in targeted middle meningeal artery devascularization for chronic subdural hematomas (CSDH) and as a critical preoperative or radiosurgical adjunct for complex brain arteriovenous malformations (bAVMs) and hypervascular tumors. Across these established paradigms, overcoming the profound physiological stressors associated with high-flow shunt occlusion, such as normal perfusion pressure breakthrough, dictates that technical angiographic success must be inexorably linked with rigorous, multidisciplinary neurocritical oversight.

However, contemporary practice is approaching the mechanical and biological limitations of inert liquid and particulate agents. Looking forward, the discipline is poised for a profound paradigm shift. While the development of stimuli-responsive ‘smart’ biomaterials, extracellular matrix scaffolds, and localized molecular therapies represents a theoretical shift toward active, targeted biological vascular remodeling, it is critical to distinguish these nascent concepts from current clinical benchmarks. Although these emerging strategies show immense promise in preclinical models and early-phase translational research, their definitive translation to standardized human neurointervention remains the next major hurdle. When these bioactive platforms are eventually integrated with the ultra-distal navigation capabilities and hemodynamic stability offered by investigational endovascular robotic systems, they hold the transformative potential to move the field beyond physical vessel occlusion, actively guiding localized cellular healing and redefining the future of precision neurointervention.

## Figures and Tables

**Table 1:** Choosing the Right Multimodal Approach for bAVMs.

Factor	Preferred Approach	Rationale & Reported Evidence
Small bAVM (<3 cm, SM Grade I–II)	SRS alone or microsurgery	High cure rates (>90%) with minimal surgical morbidity [30]
Medium bAVM (3–4 cm, SM Grade III)	Preoperative embolization + Microsurgery ± SRS for residual AVM	Preoperative embolization devascularizes the nidus; SRS treats eloquent microsurgical remnant [9,30,31]
Large bAVM (>4 cm, SM Grade IV–V)	Embolization (Onyx™, PHIL™, or n-BCA) + SRS, or staged SRS	Volume reduction for SRS optimization. Decreases the target diameter to improve the likelihood of total nidal obliteration [10,32,33]
Deep bAVMs (Brainstem, Basal Ganglia)	SRS first, surgery if needed	Minimizes iatrogenic injury to brainstem or basal ganglia [30,34]
Ruptured bAVM	Microsurgery (if accessible), or embolization (Onyx™, PHIL™, or n-BCA) + SRS	Priority on hematoma evacuation and immediate protection against re-bleeding [30,35,36]
High-flow bAVM	Embolization (Onyx™, PHIL™, or n-BCA) → Surgery or SRS	Reduces nidal turgor and intraoperative blood loss [30,31]

Data Provenance Statement: Multimodal treatment workflows and preferred approaches represent an author-derived clinical synthesis based on established Society of NeuroInterventional Surgery (SNIS) practice guidelines and Spetzler-Martin grading principles. All reported cure rates and clinical efficacy metrics (e.g., >90% for Grade I–II lesions) are directly extracted from the cited clinical series and guidelines [9,10,30,31,32,33,34,35,36]. Abbreviations: bAVM, brain arteriovenous malformation; n-BCA, n-butyl cyanoacrylate; PHIL, Precipitating Hydrophobic Injectable Liquid; SM Grade, Spetzler-Martin Grade; SRS, stereotactic radiosurgery.

**Table 2:** Illustrative Clinical Scenarios and Synthesized Evidence in dAVF Management.

dAVF Type (Anatomy)	Presentation & Angiographic Features	Intervention	Rationale for Multimodality	Reported Outcome & References
Tentorial dAVF (Cognard IV)	Headache, ataxia; tentorial feeders; single deep draining vein; cortical venous reflux	Transarterial Onyx™ embolization → Microsurgical disconnection → SRS for residual	Embolization reduces flow; surgery provides immediate cure; SRS treats tiny deep remnant	High obliteration rate reported at 12 month follow-up [45]
Anterior cranial fossa/ethmoidal dAVF (Cognard III/IV)	SAH; ophthalmic/ethmoidal feeders; direct cortical venous drainage	Attempted embolization (unsafe) → Primary microsurgical disconnection	Ethmoidal feeders, risk to ophthalmic artery/vision; surgery offers immediate definitive cure	High immediate angiographic cure rates (>95% reported in meta-analysis) [46]
Transverse-sigmoid sinus dAVF (Cognard I–IIb)	Pulsatile tinnitus, partially functional sinus involved	Transvenous coil + Onyx™ → SRS for small residual	Transvenous embolization safe & highly effective; SRS only for persistent tiny nidus	Cure with symptom resolution [39,44]
Cavernous sinus dAVF (indirect CCF; Cognard I–IIa)	Chemosis, CN VI palsy; drainage via ophthalmic veins	Transvenous coil embolization → Optional TA Onyx™ → SRS if residual	Transvenous route is first-line; surgery rarely needed; SRS for small residuals	High rate of clinical and ophthalmic recovery reported within 1–3 months [44,47]
Recurrent complex dAVF with multiple feeders (Cognard III/IV)	Recurrent symptoms after 2 prior embolizations; new pial recruitment	Targeted PHIL™ embolization → Microsurgical draining vein disconnection → SRS for scarred sinus wall remnant	Combined therapy prevents further recruitment; each modality addresses different anatomic components	Durable long-term angiographic cure reported across combined-modality series [43,47]

Data Provenance Statement: This table presents illustrative scenarios based on a clinical synthesis of reported literature; individual patient outcomes should not be inferred from these synthesized benchmarks. Detailed clinical scenarios, presentation features, and sequential intervention workflows represent an author-derived clinical synthesis based on established neurointerventional practice guidelines and anatomical access feasibility. All reported clinical timelines, symptom resolutions, and angiographic obliteration outcomes are directly extracted from the cited multicenter clinical series, systematic reviews, and practice guidelines [38,42,43,44,45,46,47]. Abbreviations: CCF, carotid-cavernous fistula; CN, cranial nerve; dAVF, dural arteriovenous fistula; PHIL, Precipitating Hydrophobic Injectable Liquid; SAH, subarachnoid hemorrhage; SRS, stereotactic radiosurgery; TA, transarterial.

**Table 3:** Roles of Embolization in Multimodal Treatment Approaches for Intracranial Tumors.

Tumor Type	Preferred Agent(s)	Acceptable Alternatives	Advantages	Limitations	Reported Efficacy & References
Meningioma	Liquid embolics (Onyx™, n-BCA)	PHIL™, Squid™, or PVA (larger particles)	Deep tumor bed penetration for preoperative devascularization or standalone tumor necrosis.	Dangerous anastomoses and pial supply risks; time-sensitive liquid polymerization; transient inflammatory response	80–100% occlusion [21,22,53]; significantly reduced blood loss [11]; 1–4% deficit rate [11,21]
Paraganglioma	PVA particles for pre-op devascularization; coils for large ECA feeders; Onyx™ or n-BCA used for deeper penetration when needed	Squid™, PHIL™ (liquid agents)	Effective ECA feeder control; distal penetration in high-flow lesions via liquid agents	Cranial nerve ischemia; superselective catheterization required; strict flow control needed with liquids	Effective preoperative devascularization of ECA feeding pedicles with low procedural complication rates [50]
Juvenile nasopharyngeal angiofibroma (JNA)	PVA for small-to-medium arteries and Onyx™ for deeper penetration	Coils for large arteries; n-BCA in selected cases; Squid™/PHIL™ used by pressure-cook/stop-flow techniques	Excellent distal filling; long-lasting occlusion in extensive lesions; optimized surgical control	ICA/ophthalmic anastomoses risks; superselective technique required; increased procedural time and radiation with Onyx™	90–100% success [54]; drastically minimizes intraoperative hemorrhage [50,54]
Renal cell carcinoma (RCC) metastasis	Onyx™ or n-BCA for intratumoral penetration; PVA for ECA feeders; coils for large direct feeders/shunts	PHIL™/Squid™	Durable devascularization; deep nidal filling; effective for direct shunts and large arterial inflow	Venous occlusion; pulmonary embolus; non-target embolization risks	High success; effective for pre-op devascularization or palliation [55]
Melanoma metastasis	n-BCA/Onyx™ when intratumoral arterial supply allows; PVA can be used for superficial ECA feeders	Coils for large feeders; PHIL™ as alternative	Targeted microvascular penetration; durable occlusion; effective for preoperative devascularization	Variable arterial supply limits efficacy; high non-target embolization risk with pial recruitment	Case-dependent efficacy; primarily utilized for targeted palliation [52]

Data Provenance Statement: Preferred embolic agent selections, acceptable alternatives, and procedural advantages represent an author-derived clinical synthesis based on neurovascular material science principles and tumor angioarchitecture. All reported occlusion percentages, intraoperative blood loss reductions, and procedural morbidity rates (e.g., 80–100% occlusion and 1–4% deficit rate in meningiomas) are directly extracted from the cited clinical series and matched-cohort studies [11,21,22,49,50,51,52,53,54,55]. Abbreviations: ECA, external carotid artery; ICA, internal carotid artery; JNA, juvenile nasopharyngeal angiofibroma; n-BCA, n-butyl cyanoacrylate; PHIL, Precipitating Hydrophobic Injectable Liquid; pre-op, preoperative; PVA, polyvinyl alcohol; RCC, renal cell carcinoma.

## Data Availability

Data sharing is not applicable to this article as no new raw data were created or analyzed in this study. All clinical narratives, procedural outcomes, and conceptual frameworks discussed in this review are derived from the previously published, publicly available literature, which is comprehensively cited within the reference list.

## Abbreviations

ACVRL1	Activin A Receptor Like Type 1
AI	Artificial Intelligence
Ang-2	Angiopoietin-2
AP	Anteroposterior
AVM	Arteriovenous Malformation
bAVM	Brain Arteriovenous Malformation
BMP	Bone Morphogenetic Protein
BMP10	Bone Morphogenetic Protein 10
BRAF	B-Raf Proto-Oncogene, Serine/Threonine Kinase
CCF	Carotid-Cavernous Fistula
CFD	Computational Fluid Dynamics
CN	Cranial Nerve
CSDH	Chronic Subdural Hematoma
CT	Computed Tomography
CVR	Cortical Venous Reflux
dAVF	Dural Arteriovenous Fistula
DMSO	Dimethyl Sulfoxide
DSA	Digital Subtraction Angiography
ECA	External Carotid Artery
ECM	Extracellular Matrix
EMBOLISE	Embolization of the Middle Meningeal Artery With Onyx Liquid Embolic System in the Treatment of Subacute and Chronic Subdural Hematoma
ENG	Endoglin
EVD	External Ventricular Drain
EVOH	Ethylene Vinyl Alcohol
FDA	U.S. Food and Drug Administration
HHT	Hereditary Hemorrhagic Telangiectasia
HIF	Hypoxia-Inducible Factor
ICA	Internal Carotid Artery
ICH	Intracranial Hemorrhage
ILT	Inferolateral Trunk
JNA	Juvenile Nasopharyngeal Angiofibroma
KRAS	Kirsten Rat Sarcoma viral oncogene homolog
MeSH	Medical Subject Headings
MHT	Meningohypophyseal Trunk
MMA	Middle Meningeal Artery
MMAE	Middle Meningeal Artery Embolization
MTA	Medial Tentorial Artery
n-BCA	n-butyl cyanoacrylate
NPPB	Normal Perfusion Pressure Breakthrough
PCA	Posterior Cerebral Artery
PEG	Polyethylene Glycol
PGLA	Poly(glycolide-co-lactide)
PHIL	Precipitating Hydrophobic Injectable Liquid
pre-op	Preoperative
PVA	Polyvinyl Alcohol
RAS-MAPK	Rat Sarcoma-Mitogen-Activated Protein Kinase
RCC	Renal Cell Carcinoma
RGD	Arg-Gly-Asp (tripeptide adhesion motif)
SAH	Subarachnoid Hemorrhage
SM Grade	Spetzler-Martin Grade
SMAD	Suppressor of Mothers against decapentaplegic transcription factor
SNIS	Society of NeuroInterventional Surgery
SRS	Stereotactic Radiosurgery
STEM	Squid Trial for the Embolization of the Middle Meningeal Artery for Treatment of Chronic Subdural Hematoma
TA	Transarterial
TAE	Transarterial Embolization
TVE	Transvenous Embolization
V	Venous drainage
VA	Vertebral Artery
VEGF	Vascular Endothelial Growth Factor
VHL	Von Hippel–Lindau
VSMC	Vascular Smooth Muscle Cell
WEB	Woven EndoBridge
YIGSR	Tyr-Ile-Gly-Ser-Arg (laminin-derived peptide)
